# CYP450 phenotyping and metabolite identification of quinine by accurate mass UPLC-MS analysis: a possible metabolic link to blackwater fever

**DOI:** 10.1186/1475-2875-12-214

**Published:** 2013-06-21

**Authors:** Sean R Marcsisin, Xiannu Jin, Theresa Bettger, Nicholas McCulley, Jason C Sousa, G Dennis Shanks, Babu L Tekwani, Rajnish Sahu, Gregory A Reichard, Richard J Sciotti, Victor Melendez, Brandon S Pybus

**Affiliations:** 1 Department of Drug Development, Division of Experimental Therapeutics, Walter Reed Army Institute of Research, Silver Spring, MD 20910, USA; 2 Australian Army Malaria Institute, Gallipoli BarracksEnoggera, 4051QLD, Australia; 3 National Center for Natural Products Research, School of Pharmacy, University of Mississippi, Oxford, MS 38677, USA

**Keywords:** Quinine, Blackwater fever, Metabolism, CYP450, Metabolite identification

## Abstract

**Background:**

The naturally occurring alkaloid drug, quinine is commonly used for the treatment of severe malaria. Despite centuries of use, its metabolism is still not fully understood, and may play a role in the haemolytic disorders associated with the drug.

**Methods:**

Incubations of quinine with CYPs 1A2, 2C9, 2C19, 2D6, and 3A4 were conducted, and the metabolites were characterized by accurate mass UPLC-MS^E^ analysis. Reactive oxygen species generation was also measured in human erythrocytes incubated in the presence of quinine with and without microsomes.

**Results:**

The metabolites 3-hydroxyquinine, 2’-oxoquininone, and O-desmethylquinine were observed after incubation with CYPs 3A4 (3-hydroxyquinine and 2’-oxoquininone) and 2D6 (O-desmethylquinine). In addition, multiple hydroxylations were observed both on the quinoline core and the quinuclidine ring system. Of the five primary abundance CYPs tested, 3A4, 2D6, 2C9, and 2C19 all demonstrated activity toward quinine, while 1A2 did not. Further, quinine produced robust dose-dependent oxidative stress in human erythrocytes in the presence of microsomes.

**Conclusions:**

Taken in context, these data suggest a CYP-mediated link between quinine metabolism and the poorly understood haemolytic condition known as blackwater fever, often associated with quinine ingestion.

## Background

Usage of the alkaloid anti-malarial agent, quinine to treat resistant strains of *Plasmodium falciparum* and as an intravenous treatment for severe malaria has increased over the past few decades [1-4]. Several *in vitro* and *in vivo* studies have reported numerous metabolites of quinine, including 3-hydroxyquinine, O-desmethylquinine, and 2’-oxoquininone and speculations have been made concerning their origins. However, the CYP 450 enzymes responsible for their formation have yet to be identified [4-8]. For example, inhibition studies have indicated that 3-hydroxyquinine production may be CYP 3A4 mediated [4,9,10]. However, Wanwimolruk *et al*. have shown that, while pretreatment with the CYP 3A4 inducer, rifampin seemed to enhance quinine clearance, no appreciable changes in 3-hydroxyquinine formation were noted after such treatment [4,8]. In order to address some of these inconsistencies in the literature, a set of *in vitro* experiments were undertaken to unambiguously assign the metabolites of quinine produced by each of the five primary abundance CYPs (1A2, 3A4, 2C9, 2C19, and 2D6).

Quinine treatment is associated with several adverse events which may have a significant metabolism component. One of the least understood and most severe is blackwater fever (BWF), a haemolytic disorder characterized by massive amounts of haemoglobin in the urine. BWF has long been associated with falciparum malaria and irregular quinine ingestion [11]. Although quinine is not traditionally thought of as an oxidant drug, recent data suggest a link between quinine ingestion, glucose-6-phosphate dehydrogenase (G6PD) deficiency, malaria infection, and the occurrence of BWF [12,13]. Further, Bloom *et al*. reported that after incubation with microsomes and quinine, reduced glutathione (GSH) depletion was comparable to that of primaquine [14]. It is reasonable to assume that the metabolic formation of quinine, quinolones, could add low levels of oxidative stress, which combined with the background oxidative environment of a parasitized G6PD-deficient erythrocyte, may lead to the haemolytic response associated with BWF. To this end, attempts to identify novel metabolites, quinolone or otherwise, which could produce oxidative stress were also made in the present work.

## Methods

### Chemicals used

Chemicals used were: quinine (Sigma, St Louis, MO, USA, # 6119-47-7), nicotinamide adenine dinucleotide phosphate, oxidized form (NADP) (Sigma, # 077K7000), acetonitrile (Fisher Scientific, Waltham, MA, USA, #972970), glucose-6-phosphate (G6P) (Sigma, # 046K3779), glucose-6-phosphate dehydrogenase (G6PD) (Sigma, # 068K3795), and magnesium chloride (MgCl_2_) (Sigma, #102K0154). Mobile phases were made with HPLC grade water, acetonitrile, and formic acid. The ROS probe 2,7-dichlorofluorescein diacetate (DCFDA) was obtained from Molecular Probes/Invitrogen, (Eugene, OR, USA).

### CYP incubations

*In vitro* metabolism studies with isoenzymes were conducted according to the manufacturer’s instructions (BD Gentest, San Jose, CA, USA). Briefly, the procedure was as follows. A 30 μl aliquot of 5 mg/ml isoenzyme, either CYP 1A2, 3A4, 2C9, 2C19, or 2D6, was mixed with NADPH regeneration system A (50 μl) and B (10 μl), and 990 ml phosphate buffer (pH 7.4, 100 mM) was added. The solution was mixed gently by pipetting and incubated at 37°C for about 2 min, and then the test compound, quinine (10 μM final concentration), was added. A portion of the mixture (120 μl) was then collected at several time points (0, 60 min) followed by quenching with an equal volume of acetonitrile. The samples were vortexed for 30 sec, and centrifuged at 13,600 *g* at 4°C for 10 min. Supernatant was collected and loaded onto 96-well plates (200 μl/well) for LC-MS analysis.

### Accurate mass metabolite identification

Quinine samples were analysed using a Waters (Milford, MA, USA) Acquity UPLC system coupled to a Xevo Q-ToF mass spectrometer equipped with a standard electrospray ionization source. Chromatographic separations were achieved using a Waters Acquity BEH C18 1.7 μm 2.1 mm x 100 mm column with a 2 to 98% acetonitrile gradient over 6.10 min at a flow rate of 0.70 mL/min. Mobile phase A consisted of 10 mM ammonium bicarbonate and mobile phase B consisted of acetonitrile. The gradient consisted of phase B increasing from 2 to 60% in the time period of 0 to 2.9 min, followed by 60 to 98% from 2.9 to 4.7 min, holding at 98% B from 4.7 to 5.2 min, and then returning to 2% B from 5.2 to 6.1 min. MS conditions were optimized for quinine detection in the positive electrospray mode with the corresponding instrumental parameters: capillary 3 kV, sampling cone 35 V, extraction cone 4 V, source temperature 120°C, desolvation temperature 550°C, cone gas flow 30 L/Hr, and desolvation gas flow 1,000 L/hr. Low energy MS scans were conducted using a collision energy of 6 V. Quinine fragments were produced using the MS^E^ mode with a collision energy ramp from 30–35 V. Quinine metabolites were indentified and analysed using Waters Metabolynx software, MS^E^, and MS/MS analysis.

### Reactive oxygen species (ROS) formation kinetics assay

The intra-erythrocytic formation of ROS was monitored in real-time with 2’7’- dichlorofluorescein diacetate (DCFDA), a fluorescent ROS probe as described earlier [15]. Human erythrocytes that had been collected in citrate phosphate anticoagulant were used. The erythrocytes were washed twice with 0.9% saline and suspended in PBSG at a haematocrit of 10%. A 60 mM stock of DCFDA was prepared in DMSO and added to the erythrocytes suspension to obtain the final concentration of 600 mM. The suspension containing 600 mM of DCFDA was incubated at 37°C for 20 min and centrifuged at 1,000 g for 5 min. The pellet of DCFDA-loaded erythrocytes was suspended in PBSG to 50% haematocrit and used for kinetic ROS formation assay. The microsomal metabolism-linked assay was directly set up in a clear flat-bottomed, 96-well microplate. The reaction mixture contained 40 ml of DCFDA-loaded erythrocytes, 40 ml NADPH regeneration cocktail (0.8 mmol NADP^+^, 5 mmol G6P, 3 mmol MgCl_2_ and 0.2 units G6PD), 40 ml KCl (31 mmol), 10 ml of pooled human liver microsomes (25 pmol CYP content), 2 ml of quinine (concentration as presented below) and potassium phosphate buffer (100 mM, pH 7.4), to make up the final volume to 200 ml. The controls without drug (with and without microsomes) were also set up simultaneously. Each assay was set up at quadruplicate. The plate was immediately placed in a microplate reader programmed to kinetic measurement of fluorescence (excitation 488 nm and emission 535 nm) for 2 hr with 5-min time intervals. The results are presented as increase in time-dependent fluorescence by quinine with and without microsomes.

## Results

**Figure 1:**
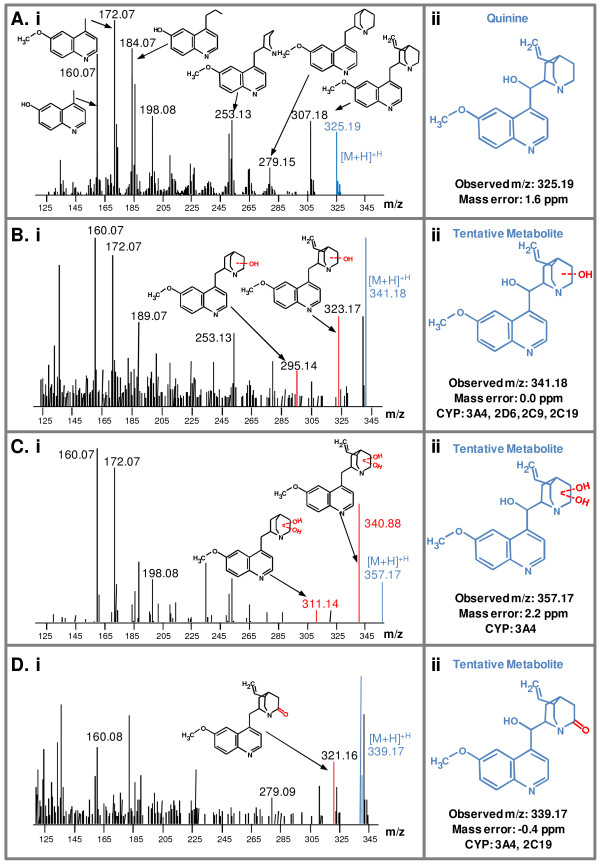
**MS**^**E **^**identification of quinine quinuclidine ring metabolites.** Shown in panels **A**-**D** are the corresponding MS^E^ spectra (i) for quinine and each quinine quinuclidine ring metabolite. The *m*/*z* values for each ion are indicated as well as the assigned quinine fragment. The assigned metabolite structures are shown in (ii). The observed parent ion *m*/*z*, mass error, and CYPs responsible for production of each metabolite are indicated under each structure.

**Figure 2:**
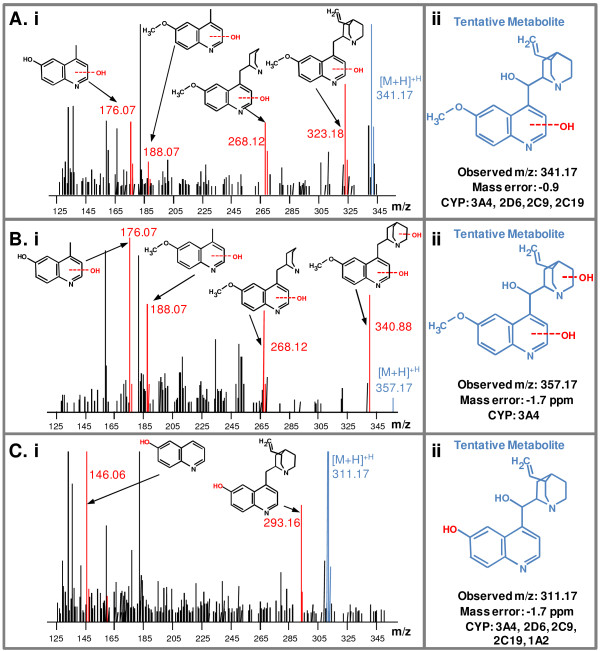
**MS**^**E **^**identification of quinine quinoline core metabolites.** Shown in panels **A**-**D** are the corresponding MS^E^ spectra (i) for each quinine quinoline core metabolite. The *m*/*z* values for each ion are indicated as well as the assigned quinine fragment. The assigned metabolite structures are shown in (ii). The observed parent ion *m*/*z*, mass error, and CYPs responsible for production of each metabolite are indicated under each structure.

In order to determine the specific metabolites likely to be formed physiologically by each of the five primary abundance CYPs (1A2, 3A4, 2C9, 2C19, and 2D6), 1-hr incubations with quinine were conducted and the subsequent metabolites formed were analysed by accurate mass UPLC-MS^E^. Table 1 summarizes the metabolites observed with each CYP and Figures 1 and 2 show the corresponding MS^E^ spectra for each metabolite. In general, four types of metabolites were observed; hydroxylations on the quinoline core, hydroxylations on the quinuclidine ring, demethylations, and ketones. Further, at the concentrations chosen in this study, 3A4 metabolized quinine most extensively, followed by 2C19, 2C9, and 2D6. CYP 1A2 showed very little appreciable activity towards quinine, with only trace amounts of a demethylated metabolite being observed after 60 min. Assignments of localization for each biotransformation to either the quinoline core or quinuclidine ring were made by MS^E^ fragmentation. Specific variants of each subtype of metabolite observed are discussed in more detail below.

**Table 1:** **Quinine metabolites formed upon CYP 3A4**, **2D6**, **2C19**, **2C9 and 1A2 incubation Shown are the metabolites observed with each corresponding CYP**

	**Metabolite observed**					**MS information**		**Relative % metabolite present**				
**Metabolite**	**3A4**	**2D6**	**2C19**	**2C9**	**1A2**	***m***/***z***	**T**_**r**_ (**min**)	**3A4**	**2D6**	**2C19**	**2C9**	**1A2**
Parent	+	+	+	+	+	325.19	3.6	49.04	95.19	78.22	93.90	99.31
Ketone	+	-	+	-	-	339.17	3.12	0.27	0.00	0.09	0.00	0.00
Hydroxylation	+	+	+	+	-	341.18	2.69	0.43	0.66	1.22	0.06	0.00
Hydroxylation	+	+	+	+	-	341.18	2.85	44.88	0.98	8.36	0.72	0.00
Hydroxylation	+	+	+	+	-	341.18	2.94	2.22	1.47	10.89	5.20	0.00
Hydroxylation/Ketone tautomers	+	-	+	+	+	341.18	3.14	0.32	0.00	0.01	0.01	0.06
Demethylation + hydroxylation	+	-	-	-	-	327.18	2.48	0.16	0.00	0.00	0.00	0.00
Demethylation	+	+	+	+	+	311.17	3.16	0.15	1.70	1.23	0.10	0.62
2x Hydroxlyation	+	-	-	-	-	357.18	2.57	1.18	0.00	0.00	0.00	0.00
2x Hydroxlyation	+	-	-	-	-	357.18	2.41	1.36	0.00	0.00	0.00	0.00

The plus and minus signs indicate whether the listed metabolite was observed with each CYP after 60 minute incubations. The corresponding *m*/*z* values observed and retention times (t_r_) for each metabolite are listed. The relative % of each metabolite formed is also listed.

### Modifications on the quinuclidine ring

MS^E^ analysis of the quinine metabolites detected revealed that two portions of quinine were modified: the quinuclidine ring and quinoline core. This section will focus on the quinuclidine ring and the results for each ring metabolite are illustrated in Figure 1. The MS^E^ fragmentation pattern of unmodified quinine is illustrated in Figure 1A and shows the different fragmentations of quinine produced upon MS^E^ fragmentation. These fragments encompassed the entire molecule and allowed for the localization of chemical modifications to quinine.

After incubation with CYP 3A4, multiple metabolites were observed with an *m*/*z* of 341.18. As represented in Table 1, the predominant metabolite was observed at 2.85 min. MS^E^ fragmentation showed mass changes of + 16 Da to quinuclidine ring fragments which was consistent with the addition of a hydroxyl group (Figure 1B). Given the relative intensity of this metabolite, it was tentatively assigned as 3-hydroxyquinine. This metabolite was observed to a lesser extent after incubation with CYPs 2C9, 2C19, and 2D6. Two other metabolites with the same observed *m*/*z* (341.18), retention times of 2.69 and 2.94 min, and MS^E^ fragmentation consistent with the addition of a hydroxyl group on the quinuclidine ring (as described above) were noted after incubation with all CYPs tested except 1A2 (data not shown). In addition to single hydroxylations of the quinuclidine ring, incubations with CYP 3A4 also resulted in the appearance of a metabolite with retention time of 2.41 min and an observed *m*/*z* of 357.18. The + 32 Da shift was localized to quinuclidine ring fragments as illustrated in Figure 1C.

In addition to mass changes consistent with hydroxylations, one metabolite appeared at 3.12 min with an observed *m*/*z* of 339.17. This metabolite appeared with CYP 3A4 and 2C19 quinine incubations. The + 14 Da mass increase is consistent with the addition of a hydroxyl followed by desaturation to the corresponding ketone. The MS^E^ fragmentation pattern for this metabolite is shown in Figure 1D and indicates a mass change in one quinuclidine ring fragment (*m*/*z* 321.16). This fragmentation pattern is consistent with a ketone-forming alpha to the nitrogen of the quinuclidine ring. Several other metabolites with an *m*/*z* of 339.17 were observed, however, these were not of ample intensity to make a definitive determination as to the location of these modifications. It should be noted that this ketone is unlikely to be on the quinoline core as this modification would have an expected *m*/*z* of 341.18. The ketone formation observed must occur somewhere on the quinuclidine ring.

### Modifications on the quinoline core

The quinoline core of quinine was also modified upon incubation with the various CYPs. Incubations with either CYP 3A4, 2C19, or 2C9 resulted in the appearance of a metabolite with a retention time of 3.06 min and an observed *m*/*z* of 341.17. The MS^E^ fragmentation pattern for this metabolite is shown in Figure 2A and the +16 Da mass shift is consistent with the addition of a hydroxyl group to the quinoline core. This metabolite was tentatively assigned as the 2’ hydroxylated form of quinine. It should be noted that by accurate mass and MS^E^ fragmentation, this metabolite is indistinguishable from its expected tautomer (2’–oxoquininone). Further, a metabolite appeared after CYP 3A4 incubation at a retention time of 2.57 min with an *m*/*z* of 357.17 consistent with the addition of two hydroxyl groups (+ 32 Da). MS^E^ fragmentation patterns indicated that one of these hydroxylations occurs on the quinoline core, with the other occurring on the quinuclidine ring as shown in Figure 2B. In addition to the hydroxylated metabolites described above, all CYPs tested generated a metabolite with a retention time of 2.48 min and an observed *m*/*z* of 311.17. This peak was assigned to the structure in which the 6’ methoxy has been demethylated (- 14 Da). MS^E^ fragmentation of this metabolite is shown in Figure 2C and confirms the loss of a methyl group to all fragments associated with the quinoline core. Although CYP 2D6 generated the most of this metabolite under these experimental conditions, all CYPs tested generated detectable amounts of this metabolite. It should be noted that the primary purpose of the present study was to identify the number and type of CYPs involved in quinine clearance and not to attribute relative contributions from each *in vivo*. Further, as with 2’ hydroxylation, this metabolite is indistinguishable by accurate mass or MS^E^ fragmentation from its expected tautomer (6’-oxoquininone).

### ROS formation kinetics assay

**Figure 3:**
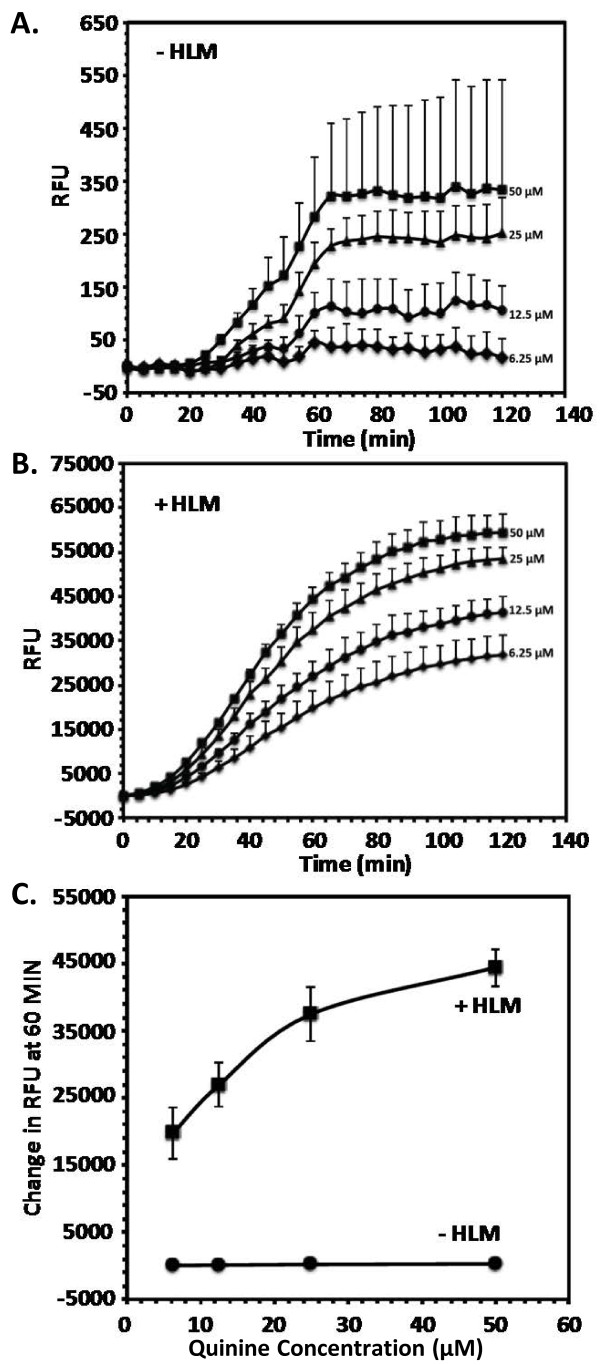
**Time**/**Concentration**-**dependent oxidative stress produced in normal human erythrocytes by microsomal metabolites of quinine *in vitro.*** Plotted are the changes in relative fluorescence units (RFU) for various quinine concentrations as a function of time **A**.) without human liver microsomes (HLM) and **B**.) with HLM. **C**.) Concentration dependence of ROS generation at 60 min is plotted. Each point represents values mean ± SD of four observations.

Having established from CYP incubations and MS analysis that quinine forms metabolites that have the potential to redox cycle and create oxidative stress, the ability of quinine to produce ROS was assayed and the results illustrated in Figure 3. Quinine was assayed in normal human erythrocytes in the presence and absence of pooled human liver microsomes. Quinine did not produce noticeable oxidative stress in normal human erythrocytes without microsomes as indicated by no significant increase in DCFDA fluorescence. However, incubation of human erythrocytes with quinine in presence of human liver microsomes produced robust reactive oxidative stress as indicated by time-dependent increase in DCFDA fluorescence. These observations indicate that quinine metabolites generated through CYP-mediated pathways are responsible for producing intracellular oxidative stress in the erythrocytes. Further, the increase in ROS production by the microsomal metabolites of quinine was observed to be concentration dependent (Figure 3).

## Conclusions

The cinchona alkaloid anti-malarial agent, quinine is extensively metabolized *in vivo*, and many of the resulting metabolites have been reported both in urine and plasma [4,7,8,16]. While these studies have largely used synthetic metabolites of the diasteriomer quinidine as reference standards for HPLC identification rather than direct observation via accurate mass MS, the consensus assignments are not in question. However, ambiguity remains concerning the specific CYP pathways responsible for the production of the major metabolites, and therefore the primary clearance routes of this drug. For example, Wanwimolruk *et al*. observed that both cigarette smoking and rifampin pretreatment enhance the clearance of quinine, suggestive of important roles for both CYP3A and 1A families in quinine clearance. They further noted that neither observation correlated with a significant increase in the ratio of 3-hydroxyquinine, a major metabolite, in the urine [4,8]. Other metabolites have been observed in urine such as 2’-oxoquininone and O-desmethylquinine which have not been assigned to specific CYP pathways [6,7,16].

**Figure 4:**
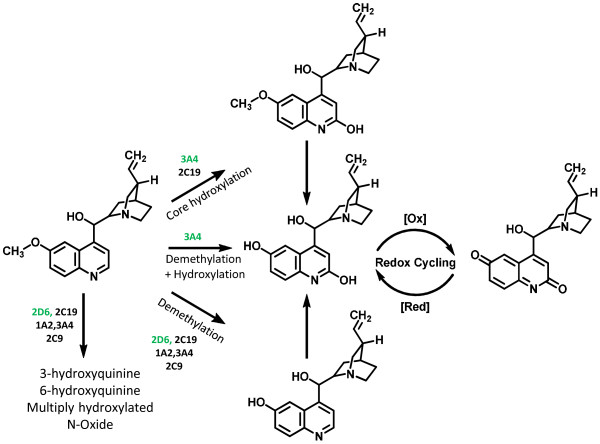
**Summary of quinine metabolism.** Shown are the identified metabolites of quinine and their predicted redox active forms. The CYPs responsible for each transformation are indicated for each pathway and the CYPs primarily responsible for each transformation are indicated in green. Several other metabolites are listed for which the structures are not shown (3-hydroxyquinine, 6-hydroxyquinine, N-oxide, and multiply hydroxylated).

In the present study, CYP 3A4 was identified as predominantly responsible for the production of 3-hydroxyquinine, and a second hydroxylation localized to the quinoline core. To date, the only hydroxylation reported on the quinoline core occurred at the 2’ position, therefore this metabolite most likely corresponds to 2’-hydroxyquinine. It is important to note that the tautomeric quinolone form of this metabolite is isobaric, but is assumed to exist for this metabolite and all others in which hydroxylation occurs on the quinoline core. Three other keto metabolites were observed after incubation with CYP 3A4, one of which localized to the quinuclidine ring, as determined by MS^E^, and the others which had fragmentation patterns with insufficient signal intensity for accurate localization. However, the *m*/*z* associated with each of these ketone metabolites was only consistent with addition to the quinuclidine ring. CYP3A4 was also found to mediate the production of two doubly hydroxylated metabolites, one in which hydroxylation occurred both on the quinoline core and the quinuclidine ring (2’,3-dihydroxyquinine is the most likely possibility for assignment) and a second with two hydroxylations occurring on the quinuclidine ring. Given the abundance in plasma of 3-hydroxyquinine, it is reasonable to assume that hydroxylation at the 3 position accounts for one of these, while the other hydroxylation may occur alpha to the nitrogen at the 6 position on the quinuclidine ring. This would correspond to the reported dihydroxy non-phenolic metabolite reported by Brodie *et al*. [16]. While O-demethylation was observed after incubation with all five CYPs tested, under these experimental conditions CYP 2D6 produced the most of this metabolite followed by 2C19, 3A4, 1A2, and 2C9 respectively. Further, CYP 3A4 was found to catalyze hydroxylation of this metabolite, presumably at the 2’ position for the reasons discussed above. Lastly, two other hydroxylated species were observed, which were generated at various levels with CYPs 3A4, 2D6, 2C9, and 2C19. As stated above, one of these would presumably be the substitution alpha to the quinuclidine nitrogen atom reported by Brodie *et al*., while the other would seem to be an as yet unidentified metabolite [16]. A schematic representation of the major phase I metabolic pathways elucidated from this work is provided (Figure 4).

Bloom *et al*. reported GSH depletion after incubation with microsomes and quinine, comparable to levels of known oxidant drugs [14]. Interestingly, recent data suggest quinolone drugs have the capacity to generate reactive oxygen species in the presence of human liver microsomes, and in fact have been shown to reduce glutathione levels in rat liver [17,18]. Fluoroquinolone drugs are also often associated with a haemolytic anaemia which may in some ways be similar to the haemolysis seen with blackwater fever [19-23]. Further, a strong correlation between BWF and G6PD deficiency has been established [12,13]. Oxidant molecules cause accelerated generation and accumulation of reactive oxygen intermediates (superoxide radical, hydroxyl radical and hydrogen peroxide) in the erythrocytes [24]. In the present study, it was clearly shown that products of CYP-mediated quinine metabolism generate robust, dose-dependent oxidative stress in human erythrocytes (Figure 3). This may in part be due to redox cycling of the quinone species observed after incubation with CYP 3A4 (Figure 4). This would become increasingly important in individuals with already high backgrounds of oxidative stress, such as those infected with malaria or with a genetic deficiency such as G6PD deficiency. It is reasonable to speculate that quinine metabolites rather than quinine itself are at least partially responsible for the haemolytic oxidative stress associated with BWF. While the pathogenesis of BWF is incompletely understood, the metabolite identification outlined here identifies a class of molecules that warrant further investigation, specifically regarding the ability of these species to generate oxidative stress in the erythrocyte.

## Abbreviations

BWF: Blackwater fever; CYP: Cytochrome P450; DCFDA: 2,7-Dichlorofluorescein diacetate; HLM: Pooled human liver microsomes; MS: Mass spectrometry; PBSG: Phosphate buffered saline with glucose; RFU: Relative fluorescence units; ROS: Reactive oxygen species; UPLC: Ultra performance liquid chromatography.

## Competing interests

The authors have declared that they have no competing interests.

## Authors’ contributions

SRM, XJ, GDS, JCS, VM and BSP participated in research design; SRM, XJ, TB, NM, BLT and RS conducted experiments; VM contributed new reagents or analytic tools; SRM, XJ and BSP performed data analysis; SRM, XJ, GDS, JCS, BLT, RS, GAR, RJS and BSP wrote or contributed to the writing of the manuscript. All authors read and approved the final manuscript.
